# “Can community level interventions have an impact on equity and utilization of maternal health care” – Evidence from rural Bangladesh

**DOI:** 10.1186/1475-9276-12-22

**Published:** 2013-04-02

**Authors:** Zahidul Quayyum, Mohammad Nasir Uddin Khan, Tasmeen Quayyum, Hashima E Nasreen, Morseda Chowdhury, Tim Ensor

**Affiliations:** 1 Health Economic Research Unit, University of Aberdeen, Foresterhill, Aberdeen, AB25 2ZD, UK; 2 Research and Evaluation Division, Brac. 75 Mohakhali, Dhaka, Bangladesh; 3 Health, Nutrition and Population Programme, Brac. 75 Mohakhali, Dhaka, Bangladesh; 4 Leeds Institute of Health Sciences, Charles Thackrah Building, University of Leeds, 101 Clarendon Road, Leeds, LS2 9LJ, UK

## Abstract

**Background:**

Evidence from low and middle income countries (LMICs) suggests that maternal mortality is more prevalent among the poor whereas access to maternal health services is concentrated among the rich. In Bangladesh substantial inequities exist both in the use of facility-based basic obstetric care and for home births attended by skilled birth attendant. BRAC initiated an intervention on Improving Maternal, Neonatal, and Child Survival (IMNCS) in the rural areas of Bangladesh in 2008. One of the objectives of the intervention is to improve the utilization of maternal and child health care services among the poor. This study aimed to look at the impact of the intervention on utilization and also on equity of access to maternal health services.

**Methods:**

A quasi-experimental pre-post comparison study was conducted in rural areas of five districts comprising three intervention (Gaibandha, Rangpur and Mymensingh) and two comparison districts (Netrokona and Naogaon). Data on health seeking behaviour for maternal health were collected from a repeated cross sectional household survey conducted in 2008 and 2010.

**Results:**

Results show that the intervention appears to cause an increase in the utilization of antenatal care. The concentration index (CI) shows that this has become pro-poor over time (from CI: 0.30 to CI: 0.04) in the intervention areas. In contrast the use of ANC from medically trained providers has become pro-rich (from, CI: 0.18 to CI: 0.22). There was a significant increase in the utilisation of trained attendants for home delivery in the intervention areas compared to the comparison areas and the change was found to be pro-poor. Use of postnatal care cervices was also found to be pro-poor (from CI: 0.37 to CI: 0.14). Utilization of ANC services provided by medically trained provider did not improve in the intervention area. However, where the intervention had a positive effect on utilization it also seemed to have had a positive effect on equity.

**Conclusions:**

To sustain equity in health care utilization, the IMNCS programme needs to continue providing free home based services. In addition to this, the programme should also continue to provide funding to bear the cost to those mothers who are not able to have the comprehensive ANC from medically trained providers.

## Background

There is a concern for equity in maternal health outcomes and for equity of access to maternal health services as countries progress towards the MDG 5 target of reducing maternal mortality by three quarters. Inequity undermines efforts to sustain improvements across all segments of society in accessing maternal health care services [1-3]. Evidence from low and middle income countries (LMICs) suggest that maternal mortality is more prevalent among the poor whereas access to maternal health services is concentrated among the rich [4-7]. A study based on a retrospective review of survey data for maternal health provision from 54 countries found that skilled birth attendant coverage was the least equitable indicator [8]. Addressing inequity in the use of maternal health services is important as every pregnant woman should have equal access to obstetric care for equal need. The poor face both demand and supply side barriers to health care; on the demand side, high cost of access, lack of education about services available, and cultural norms and on the supply side the constraint of inadequate facilities and lack of skilled care is common in LMICs. However, increasing the supply of quality care is necessary but not sufficient to increase the utilization of maternal health services among the poor [9-11]. To address these issues many community- based interventions have been initiated in LMICs to make maternal health care services accessible to poor and vulnerable mothers [12-14]. Community based interventions were found to be more equally distributed than those delivered in health facilities. In LMICs where most deliveries take place at home, it is vital to provide home and community- based care and outreach while promoting community mobilization to increase demand for quality, facility-based services [14]. There are examples of interventions with community based maternal health services delivery strategy which have been effective in increasing access to a range of essential maternal health services among the poor [15-19].

The maternal mortality ratio (MMR) in Bangladesh fell significantly during 1998-2010 (from 574 deaths per 100,000 live births to 194) and is progressing towards the MDG 5 target of 143/100,000 live births by 2015. However, even now more than 75% of deliveries take place at home in Bangladesh [20]. There is rural–urban inequality in the utilization of maternal health services in terms of antenatal care (ANC), facility delivery care and postnatal natal care (PNC) [11]. Previous studies suggest that the factors associated with the utilization of skilled maternal health care in rural areas are, mother’s education, socio-economic status, distance to nearest hospital, area of residence, mother’s age at birth, access to mass media and NGO and a host of other traditional factors [21-23]. Substantial inequities exist both for the use of facility-based basic obstetric care and for home births with skilled attendants [21,22,24,25]. Bangladesh has witnessed a relatively substantial expansion of maternal health interventions by both the government and non-governmental organizations in rural areas [17,26,27]. The key question remains whether these interventions have resulted in a corresponding increase in uptake of these services by poor women. There is a lack of evidence regarding the equity implications of these interventions for improving maternal health services to achieve MDG 5.

## Study methods

Bangladesh Rural Advancement Committee (BRAC), a non-governmental development organization initiated an intervention on Improving Maternal, Neonatal and Child Survival (IMNCS) in the severely poverty stricken northern part of the country. The project is based on global practices and proven interventions, contextualized to improve community maternal, new-born, and child health practices and the utilization of quality services by the poor and socially excluded. The programme was piloted in Nilphamari district in 2006 and scaled up in three other districts in 2008 [28]. The strategy uses BRAC Community Health Workers (CHWs) to create demand and provide services at community level. UNICEF and the Ministry of Health and Family Welfare also provide support by providing improved emergency obstetric and newborn care services at the facility level. The BRAC female CHWs including *Shasthaya Shebika* (SS), *Shasthya Kormi* (SK) and Newborn Health workers (NHW) provide a wide range of free domiciliary services to pregnant and breastfeeding mothers, neonates and under-five children, and refer them to public facilities if any complication arises IMNCS provides financial support for medicines, blood transfusion and transportation costs to poor pregnant mothers who cannot seek timely emergency obstetric care (EmOc) due to lack of money. The referred women are provided with full or partial reimbursement of the expenditure on such care according to their economic status.

### The IMNCS intervention

The CHWs are selected from the community with the assistance of BRAC micro-finance group members (Village Organization), people living in the community and BRAC field staff following certain criteria. The selection criteria vary for the three different cadres of CHWs. SSs are married and literate women in age group 25-45 years and are selected from the BRAC village organization. NHWs are women with similar characteristics as SS, but are required to have previous knowledge of child delivery at home. The SKs, who supervise both SS and NHW, are married women in the age-group 20-40 years and have secondary level of education. These CHWs receive basic training on: maternal, neonatal, and child health care management, referral and technical issues of field operation as required by their roles in the community. Basic training is followed by monthly and quarterly 1-day refresher trainings.

**Figure 1:**
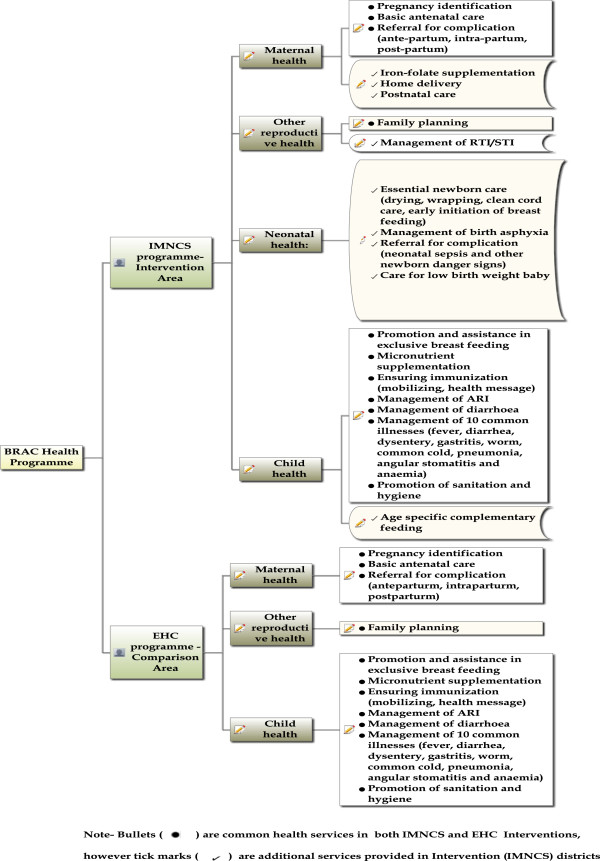
Service differences in IMNCS and EHC programmes.

The IMNCS intervention covers more intensive maternal and new-born care compared to the BRAC essential health care (EHC) programme which is well-established in many areas (see Additional file 1: Table S1, and Figure 1). In the IMNCS intervention, on average, one SS has responsibility for covering 150 households while in the comparison area one SS has to cover 300 households (Additional file 1: Table S1). There are larger numbers of CHWs (SK and NHW as well as SS) involved in the IMNCS areas compared with the control areas. This intensive community level intervention, with a greater number of trained community health workers and improved referral mechanism, is likely to improve maternal health services, e.g. number of ANC visits, antenatal and delivery care by trained providers etc. These trained community health workers identify almost all pregnancies in the intervention areas and provide maternal health services and education at home and in the community. Poor pregnant mothers who are referred to facilities in intervention areas are often also accompanied by community health workers. They also receive financial support to pay for transport and purchase of medicines. The comparison group received only essential health care including health and nutrition education, water and sanitation, family planning, immunization, pregnancy related care, vitamin-A supplementation, and basic curative services, but not the intensive maternal care and referral support as shown in Additional file 1: Table S1.

This study aims to examine whether the intervention addresses inequity in utilization of maternal health care irrespective of economic status. It is expected that there would be an increase in health care service utilization across the socio-economic groups but the question is whether this is in favour of the lower socio-economic groups which the programme attempts to achieve.

#### Study design and setting

**Figure 2:**
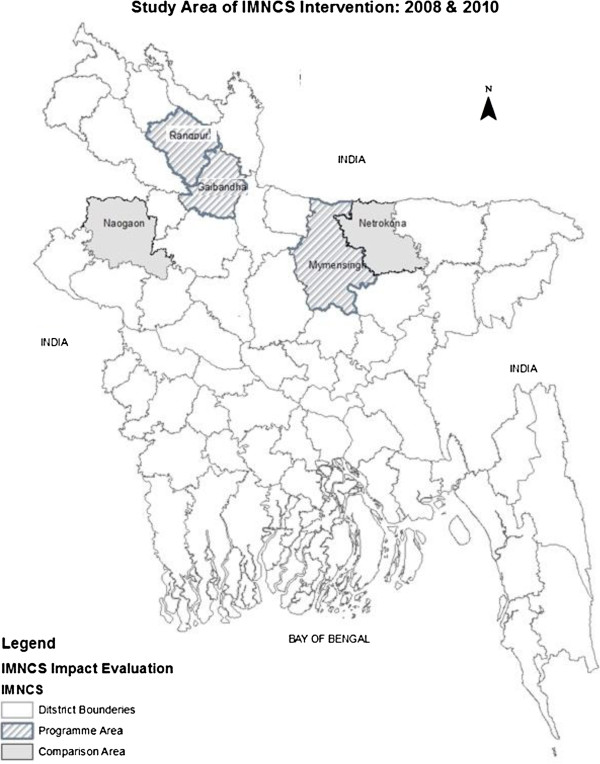
Map of study area.

This quasi-experimental study, designed as pre-post comparison, was conducted in rural areas of five districts of Bangladesh. It used information from a repeated cross sectional household survey, conducted in 2008 and 2010, on maternal health behavior in three IMNCS intervention districts and two comparison districts. The intervention districts were Gaibandha, Rangpur and Mymensingh and the comparison districts were Netrokona and Naogaon (Figure 2). The majority of the population in rural Bangladesh live below the national poverty line. Most women are home workers and look after their children. BRAC is implementing its core programmes on micro-finance, health, and education in all districts of the country (more information available at http://www.brac.net). Apart from the IMNCS programme activities noted earlier, BRAC is also involved in providing the beneficiaries with education and skill development training, creating social awareness and supporting income generating activities through the microfinance programme. The intervention districts received intensive maternity services by the community health workers who provide a wide range of services at household level.

#### Sample size and respondents

**Figure 3:**
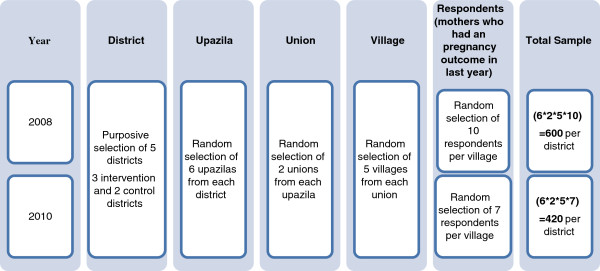
Sampling strategy.

Multi-stage random sampling was used to select the representative respondents from each district. Survey participants were randomly selected from all eligible mothers,i.e. those with a pregnancy outcome during the year preceding the survey. In 2008, the estimated sample size was 3,000 (600 per district), based on the rates of skilled delivery with a health worker and prevalence of antenatal and postnatal care. The sample was based on 80% power to detect a change of 50% (with 5% error level) and a design effect of 1.5. A non-response rate of 3% was assumed. The same sampling strategy was used in 2010, assuming an expected change in prevalence due to IMNCS intervention and the resulting sample was 2,100 respondents (420 per district, see Figure 3). The surveys were (in both 2008 and 2010) carried out on those mothers who had a pregnancy outcome in the previous year of the survey and who had a child less than one year of age at the time of survey.

Information on other health care providers such as the number of hospitals and clinics, the distance to public sector hospitals, the time needed to reach a district hospital (which can provide comprehensive obstetric care) and the number of private clinics in both intervention and comparison districts was also collected. This was done to find an exact match between intervention and comparison districts at a lower level (below sub district) applying propensity score matching (PSM).

#### Data collection

Data were collected by trained female enumerators. The data collection was monitored and cross-checked at four levels (team leaders, monitors, field supervisors and researchers). The same questionnaire and data collection procedures were applied in both 2008 and 2010. Data collection took place from September to December in 2008 (Baseline) and from September to January in 2010 (named as Midline). The survey questionnaire collected outcome information on: antenatal care (ANC) (number of visits and provider); delivery care (mode, place, provider); and postnatal care (PNC) (received within 48 hours). Explanatory variables were comprised of socio-demographic and household assets. We used data on household assets (such as owning: a TV, motor-cycle, and sewing machine) and characteristics of the households (source of drinking water, sanitation facilities and type of material used for flooring, roof and walls) to construct the wealth index applying principal components analysis (PCA) [30]. This measure was used to classify the sample households into wealth quintiles.

Ethical approval was obtained from Bangladesh Medical Research Council (BMRC) under an umbrella project “Impact Evaluation of Maternal, Neonatal and Child Health Programme of BRAC 2008-2012”. Informed verbal consent was taken from each respondent before administering the questionnaires, and the respondents name was not disclosed or used for any purpose.

### Analysis

The analysis highlights the changes in the utilization pattern of maternity care in relation to equity. To capture the changes in health care utilization over time, Difference in Differences (DiD) estimation was used. In Bangladesh for district level administration, a district is subdivided into several sub districts (*upazilas*) and an *upazila* is subdivided into a lower level of administration called *unions*. To assess the impact of the intervention, a balanced group of unions from intervention and control districts was determined using PSM. Concentration indices and rich-poor ratio were calculated in order to examine equity in utilization.

#### Difference in Differences (DiD)

Since the work by Ashenfelter and Card [31], the use of Difference-in-Differences (DiD) methods has become very widespread and has become a very popular method for estimating causal relationship. It involves identifying specific interventions and compares the differences in outcomes before and after the interventions for the region or groups affected by the intervention to the same differences for unaffected or control groups or regions. DiD here is used to compare outcomes between intervention and comparison groups before (baseline) and after (midline) the intervention [29].

DiD estimates and their standard errors have been derived from Ordinary Least Squares (OLS) in the repeated cross section surveys of data on mothers in intervention and control districts for the two periods, baseline (2008) and later period (2010). This can be shown as

$$Y_{\mathrm{ist}}=A_{s}+B_{t}+cX_{\mathrm{ist}}+\beta I_{\mathrm{st}}+{€}_{\mathrm{ist}}$$

Where *Y*_ist_ is the outcome of interest (e.g. ANC received or not, delivery with trained attendant) for the individual mothers *i* in district group *s* (e.g. intervention or control) by the time *t* (the two period) and *I*_*st*_ is the dummy for whether IMNCH intervention has affected the group *s* at time *t*. *A*_*s*_ and *B*_t_ fixed effects for the districts and years respectively, *X*_ist_ are relevant individual controls and ∈_ist_ is error term. $\hat{\beta }$ estimated by OLS indicates the impact of the intervention. The standard errors are used to determine the confidence interval.

#### Propensity score matching (PSM)

Propensity score is the probability of taking treatment given a vector of observed variables (X). If we take each union with the same propensity score, and divide them into two groups (groups with intervention and groups without intervention (T), the groups will be approximately balanced on the variables predicting the propensity score:

$$p\left(x\right)=\mathrm{Pr}\left[T=1|X=x\right]$$

where, X = observed characteristics, here *union* level characteristics (number of functioning facilities, maximum year of functioning, distance of union from *upazila* health complex, distance of district health complex from the union).

#### Concentration Index (CI) and rich-poor ratio

A concentration index (CI) provides a measure of socio-economic inequality in health care utilization. Its value varies from -1 to +1: a value close to zero indicates near equality, a value declining towards -1 indicates greater utilization among the poor (pro-poor) while a value increasing to +1 indicates greater utilization amongst wealthier group (pro-rich) . Besides a rich-poor ratio is used to show the inequality in health care utilization among the socio-economic status of the population. Household socio-economic status was classified according to the wealth quintiles constructed by ranking the households using PCA. Five groups (Q1, Q2, Q3, Q4 and Q5) were derived, where Q1 represents the poorest and Q5 the wealthiest. The rich-poor ratio is the ratio between the figures for the Q5 (wealthiest) and Q1 (poorest) for the given indicators of health services utilization.

Analyses were done with STATA Version 11.2 statistical software and we used the *concindc*[32] command for calculating the concentration index [33] to measure economic inequalities in utilization and *pscore*[34] for estimating the propensity score matching.

## Results

### Characteristics of the respondents

Characteristics of the respondents are shown in Table 1. There was no significant difference in the respondents’ age, literacy, household head, and household size between the intervention and comparison districts in both baseline and midline surveys. It was observed that the distributions of households across the wealth quintiles were significantly different in poor quintiles in comparison area.

**Table 1:** Characteristics of the respondents

	**2008- Baseline**			**2010- Midline**		
	**Intervention districts**	**Comparison districts**	**P value**	**Intervention districts**	**Comparison districts**	**P value**
Age of respondent						
<19 years	20.0	21.1	0.47	19.4	20.1	0.72
20 – 35 years	74.3	73.0	0.48	76.3	75.9	0.85
>36 years	5.8	5.9	0.92	4.3	4.0	0.76
Mean age	24.6	24.3	0.23	24.3	24.1	0.41
Married	99.4	99.1	0.54	99.2	99.3	0.84
Can read and write	51.3	54.7	0.19	57.8	58.7	0.69
Male household head	97.4	98	0.31	98.1	98.8	0.19
Mean household size	5.1	5.3	0.02	5.4	5.4	0.14
Asset quintile			0.31			0.71
Q1- poorest	19.9	23.3		17.2	24.4	
Q2	22.1	17.6		22.6	19.7	
Q3	20.6	16.9		19.9	16.7	
Q4	18.9	21.1		19.0	20.0	
Q5- richest	18.6	21.1		21.3	19.2	
N	1,484	1,046		984	676	

### Intervention impact on IMNCS utilization

The utilization rate and the possible impact (difference in differences) are presented in Table 2. This table presents the DiD with and without applying PSM. Intervention unions were matched using PSM with similar unions in the comparison group ensuring substantial overlap in their characteristics (of 60 unions 43 were in common support group). The ANC utilization (any ANC and mean number of visit) was higher in the three year exposure area compared to the comparison area. Results show that the intervention appears to have greatest impact on number of women having four or more ANC visits and it increased to 51.5% (31% after PSM) which could be attributed to the intervention. In the 2008 survey, the reasons for not seeking ANC reveal that 25.7% (Table not shown) of mothers reported that they do not feel it to be necessary and 12.1% said that they could not attend because of lack of money. After three years of the intervention, 5.2% thought ANC was not necessary and 1.6% reported that financial barriers prevented them from seeking ANC. The proportion of women receiving one or more ANC from a medically trained provider decreased in the intervention districts over time by 4.9% on total population and 1.1% with PSM correction. However, if we consider the SK to be a trained ANC provider, the impact is positive in the intervention area (Table 2).

**Table 2:** Utilization rate (%) of maternal services

	**Baseline (2008)**		**Second survey (2010)**		**Pre Difference**	**Post Difference**	**Impact**	
	**Intervention**	**Comparison**	**Intervention**	**Comparison**			**DiD**^**1**^	**DiD**^**2**^
One or more ANC	61.7	71	93.2	76.5	−9.3	16.7	26*	18.0*
Mean number of ANC visit	2.7	3	7.3	5.2	−0.3	2.1	2.4*	2.3*
4+ ANC	14.8	22.2	68.6	24.5	−7.4	44.1	51.5*	31*
ANC by medically trained provider**	38.5	53.6	34	54	−15.1	−20	−4.9*	−1.1*
ANC by trained provider***	58.5	63.2	91.4	59.5	−4.7	31.9	36.6*	30.6
**Home delivery**								
by untrained attendant	66.9	56.9	40.2	44.1	10	−3.9	−13.9*	−10*
by trained attendant	20.7	26	41	33.1	−5.3	7.9	13.2*	9.6*
**Normal Delivery at facility**								
Public hospital	4.9	6.9	8	9.2	−2.0	−1.2	0.8	1.1
Private hospital	3	2.4	2.4	1.8	0.6	0.6	0.0	.01
**C-Section Delivery**								
in Public hospital	1.4	1.4	3.4	2.4	0.0	1	1	0.0
in private hospital	3.3	6.5	5.1	9.5	−3.2	−4.4	−1.2	0.0
PNC within 48 hours	15.4	25.1	52.4	20.3	−9.7	32.1	41.8*	19*
**N**	**1,475**	**978**	**1,042**	**667**				

*Significance below 5% level; ** Medically trained provider (MTP) for ANC services includes: Doctor, Nurse, FWV, and SACMO; *** Trained provider for ANC services includes: Doctor, Nurse, FWV, SACMO and BRAC SK, 1 = Impact considering every population, the basic model, 2 = Impact after balancing with PSM.

Home delivery was dominant in all areas over time. There was a significant increase in utilizing trained attendants in the intervention compared to the comparison area and the intervention has contributed to some of the changes (DiD of 13.1). There was no significant change in the utilization of public hospital for normal delivery that can be attributed to the intervention. However, there was a significant change in the utilization of public hospitals for delivery care among the poor households (Q1) (Table 3).

**Table 3:** Utilization rate across the wealth quintiles

	**2008**						**2010**					
**Asset quintile**	**Q1**	**Q2**	**Q3**	**Q4**	**Q5**	**Total**	**Q1**	**Q2**	**Q3**	**Q4**	**Q5**	**Total**
Intervention (N)	**292**	**327**	**304**	**279**	**273**	**1,475**	**180**	**234**	**207**	**198**	**223**	**1,042**
4+ ANC (%)	6.1	7.6	15.4	15.7	31.2	14.8	62.2	65.3	67.8	70.4	76.2	68.6
**Normal Delivery at facility (%)**												
Public hospital	**3.4**	**3.7**	4.9	3.9	8.8	4.9	6.1	8.1	9.7	5.1	10.3	8.0
Private hospital	1.4	1.5	2.0	1.8	8.8	3.0	1.1	1.3	2.9	1.5	4.9	2.4
**C-Section Delivery (%)**												
in Public hospital	0.3	0.3	2.0	1.4	2.9	1.4	3.9	2.1	1.5	3.5	5.8	3.4
in private hospital	1.4	1.8	1.6	3.9	8.1	3.3	1.1	1.3	2.4	4.6	15.3	5.1
**Comparison (N)**	**229**	**171**	**164**	**208**	**206**	**978**	**163**	**131**	**112**	**132**	**129**	**667**
4+ ANC	10.9	12.1	25.9	21.6	40.4	22.2	15.8	24.8	22.1	33.3	38.5	26.5
**Normal Delivery at facility (%)**												
Public hospital	3.9	3.5	4.3	12.0	9.7	6.9	5.5	8.4	6.3	9.9	16.3	9.2
Private hospital	1.3	0.6	6.1	1.9	2.4	2.4	0.6	2.3	0.0	1.5	4.7	1.8
**C-Section Delivery (%)**												
in Public hospital	0.0	0.0	1.8	0.5	4.9	1.4	0.0	2.3	3.6	1.5	5.4	2.4
in private hospital	1.3	1.8	3.7	6.3	18.9	6.5	2.5	2.3	6.3	7.6	30.2	9.5

**Figure 4:**
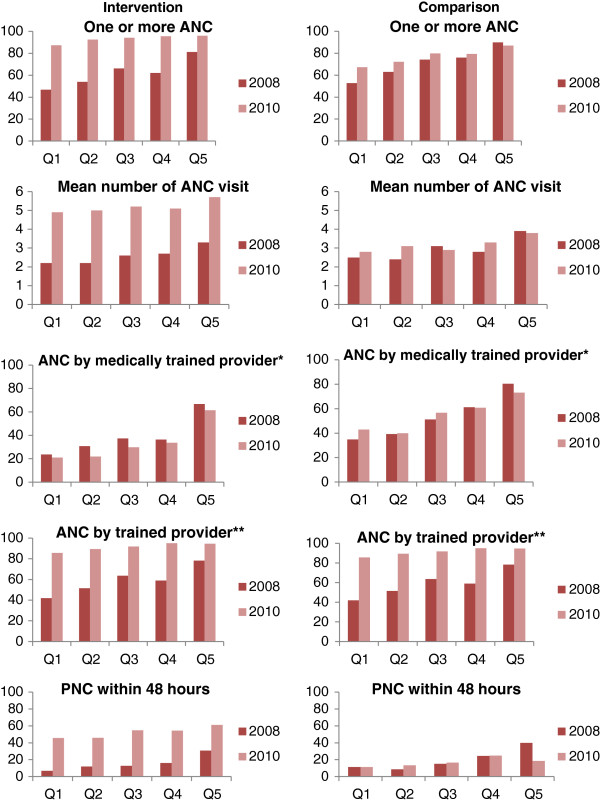
Utilization rate across the wealth quintiles.

Although no significant change can be attributed to the intervention, there is significant change in the utilization pattern among the wealth quintiles in three years exposure (Figure 4). The proportion of women receiving PNC within 48 hours increased in the intervention districts resulting in a large effect, which could be attributed to intervention (DiD: 41.8) (Figure 4).

### Equity of utilization

In this section the impact of the programme on equity is examined. Results are presented on equity of utilization in terms of concentration index and rich-poor ratio in different areas over time (Table 4). The rich poor ratio for the utilization of maternal health services have decreased in all major indicators, i.e. at least one ANC visit, more than 4 ANC, home delivery by a trained provider, C-section delivery in public facility and PNC. The rich poor ratio was calculated for the fifth (richest) quintile relative to the first (poorest) wealth quintile (Table 4). Ratios of more than one indicate that those interventions services (as a proxy measure of outcome) are used more by rich women than the poorer ones. For example, a ratio of 1.67 for delivery assistance by Medically Trained Provider (MTP) indicates that women in the richest bands of wealth were delivered by skilled providers 67% more than their poorest counterparts. The concentration index showed that, utilization of more than four ANC services has become pro-poor over time (from CI: 0.30 to CI: 0.04) in the intervention areas (Table 4). The CI showed that the uptake of ANC from a medically trained provider has become more pro-rich (from, CI: 0.18 to CI: 0.22). The uptake from medically trained provider for ANC became “pro-rich in the intervention area and pro-poor in comparison area. This is likely to be due to the situation that the poor in the intervention are now using ANC from trained provider which includes the BRAC’s trained CHWs (SK), and their services were free and mostly available at home. The CI suggests that home delivery by trained providers became more pro-poor in 2010 compared to 2008 (from CI: 0.6 to CI: -0.04). Findings also suggest that the utilization of normal delivery at public facilities also became more pro-poor (from CI: 0.17 to CI: 0.05). This pattern was not significantly evident in comparison areas (from CI: 0.24 to 0.20. The C-section delivery was found to be shifted from public facilities to private facilities in the intervention area. This could probably be due to the situation that intervention is helping the early identification of potential risk pregnancies more than it used to be. The C-section delivery in public facilities are normally more complicated than those in private facilities, as most of the private facilities refer the complicated C-section to the public facilities. The CI for utilization of PNC became pro-poor in the intervention areas (from CI: 0.37 to CI: 0.14), however, changes in CI were similar in both the areas. In Table 4, the changes in CIs between two periods are estimated simply by subtracting CI 2010 from CI 2008. The confidence intervals of the changes are calculated from standard errors of the differences in mean CIs. The standard error is computed using the following formula since these are different distributions. The formula used was : SE = square root(V1/n1 + V2/n2), where V1 and n1 are the variance and sample size of the 2008 concentration index and V2 and n2 are for 2010.

**Table 4:** Concentration index for maternal health indicator over time

			**Three years exposure to intervention**				**Comparison**			
			Rich poor ratio*	**CI**	**95% confidence interval**		Rich poor ratio*	**CI**	**95% confidence interval**	
ANC	Any ANC	2008	1.6	0.098	0.076	0.120	1.5	0.103	0.079	0.127
		2010	1.4	0.017	0.007	0.027	1	0.05	0.026	0.074
		Changes		−0.081	−0.126	−0.036		−0.053	−0.110	0.004
	ANC by medically trained provider**	2008	2.6	0.184	0.149	0.219	2.1	0.173	0.142	0.204
		2010	3.6	0.223	0.176	0.270	1.3	0.119	0.080	0.158
		Changes		0.039	−0.049	0.127		−0.054	−0.142	0.034
	ANC by trained provider***	2008	1.7	0.107	0.083	0.131	1.7	0.128	0.103	0.153
		2010	1.4	0.019	0.009	0.029	1.3	0.1	0.067	0.133
		Changes		−0.088	−0.135	−0.041		−0.028	−0.089	0.033
	4+ ANC	2008	4.8	0.307	0.244	0.370	3.4	0.254	0.191	0.317
		2010	1.5	0.039	0.015	0.063	1.9	0.169	0.100	0.238
		Changes		−0.268	−0.368	−0.168		−0.085	−0.209	0.039
Home delivery	by untrained attendant	2008	0.5	−0.072	−0.094	−0.050	0.6	−0.072	−0.103	−0.041
		2010	0.9	−0.065	−0.106	−0.024	0.4	−0.113	−0.160	−0.066
		Changes		0.007	−0.069	0.083		−0.041	−0.117	0.035
	by trained attendant	2008	1.2	0.056	−0.001	0.113	0.7	−0.073	−0.132	−0.014
		2010	0.9	−0.038	−0.079	0.003	0.4	−0.095	−0.154	−0.036
		Changes		−0.094	−0.19	0.002		−0.022	−0.118	0.074
Normal delivery	in public facility	2008	2.4	0.173	0.044	0.302	2.2	0.236	0.114	0.358
		2010	2.1	0.054	−0.062	0.170	2.3	0.204	0.069	0.339
		Changes		−0.119	−0.351	0.113		−0.032	−0.291	0.227
	in private facility	2008	6	0.384	0.221	0.547	1.7	0.114	−0.08	0.308
		2010	5.5	0.272	0.062	0.482	0.6	0.331	0.015	0.647
		Changes		−0.112	−0.473	0.249		0.217	−0.296	0.73
C-section	in public facility	2008	8	0.369	0.169	0.569	-	0.587	0.415	0.759
		2010	1.9	0.141	−0.065	0.347	-	0.358	0.142	0.574
		Changes		−0.228	−0.620	0.160		−0.23	−0.756	0.298
	in private facility	2008	5.5	0.365	0.216	0.514	1.3	0.501	0.397	0.605
		2010	17	0.513	0.397	0.629	9.7	0.508	0.4	0.616
		Changes		0.148	−0.122	0.418		0.007	−0.234	0.248
PNC	PNC within 48 h	2008	4.3	0.369	0.169	0.569	3.2	0.587	0.415	0.759
		2010	1.7	0.141	−0.065	0.347	3.3	0.358	0.142	0.574
		Changes		−0.228	−0.436	−0.02		−0.229	−0.463	0.005

* Population rich poor ratio in 2008 and 2010 (0.94, 1.02) for intervention area and (0.91, 1.24) comparison area.

**Medically trained provider for ANC services includes: Doctor, Nurse, FWV, and SACMO.

***Trained provider for ANC services includes: Doctor, Nurse, FWV, SACMO, and BRAC SK.

## Discussion

This study has attempted to assess the impact of the community based IMNCS intervention on utilization and equity of maternity services. Though the intervention was aimed at improvement of maternal, neo-natal and child health services, this study looked mainly at selected maternal health services. Significant improvements in utilization was observed in all areas except ANC provided by medically trained providers. The amount of change that could be attributed to the intervention varied and was greatest for utilization of ANC and PNC. Home delivery by trained attendant and C-section in public facility also increased considerably. Where the intervention had a positive effect on utilization it also seemed to have had a positive effect on equity.

The most significant impacts attributable to the intervention were increased utilization of ANC care and accessing PNC within 48 hours. This might be because of the door to door free services provided by the BRAC SKs. The findings are similar to a community based intervention in Burma [17]. In another intervention, the home-based skilled birth attendance (SBA) programme in Bangladesh which provided domiciliary ANC, skilled delivery care and PNC, there was a substantial increase in the utilization of ANC but very little increase in the utilization of PNC [21].

This study found that the utilization of ANC (one or more than four ANC visits) increased and became more pro-poor over time indicating that the programme can reach mothers irrespective of the wealth quintiles. However, utilization of ANC by Medically trained provider (MTP) gives an example of an unintended consequence of the programme. The proportion of women receiving ANC care from a medically trained provider decreased over time and the decline was high among the poorer quintiles. During their domiciliary ANC services CHWs encouraged mothers to obtain at least one ANC from a medically trained provider during their pregnancy. This will enable the mothers to identify potential risks in pregnancies and delivery care. In community based interventions in Tanzania [12] and Pakistan [35] the counseling by CHWs during household visits resulted in increased number of early ANC bookings and ANC visits in the health facilities. In the IMNCS intervention areas the reason for not seeking ANC services from MTP may be due to receiving several ANC visits from a BRAC CHW; women do not perceive the need to go for further care from MTP. In other studies common reasons negatively associated with seeking ANC from health facilities by poor women were found to be the distance to heath facilities, the mother’s level of education and socioeconomic status [36].

Utilization of trained providers for delivery at home in the intervention areas also increased which could be attributed to the BRAC NHWs and this increase was greatest among the poor. This result was in contrast with the findings of a study in Matlab, Bangladesh where the use of trained attendants for home delivery was found to be pro-rich [24]. However, the midwifery programme in Indonesia, with an emphasis on outreach services at the women’s home and the community skilled birth attendance programme was successful in increasing skilled attendance in birth among the poor but the access to emergency obstetric care in hospital remained neglected [16].

C-section rates in public facilities in the IMNCS intervention areas increased among the poor. The increase in C-sections indicates that the BRAC CHWs were likely to be effective in early identification of pregnancy related complications and increased referral of such cases to public hospitals [37]. Results of a study in rural Bangladesh indicate that the strongest determinant of whether a rural family uses medically trained personnel for childbirth is when delivery complications are anticipated or encountered [38]. In Tanzania, failure to plan in advance for transport was also recognized as an obstacle to receiving emergency obstetric care [39]. In addition, insufficient counseling during ANC visits at facility level can have a potentially negative effect on the utilization of skilled delivery and immediate post natal care. Studies in India and Cambodia show that women who attend ANC care are more likely to seek skilled delivery care [40,41].

The uptake of post natal care (PNC) significantly increased in the IMNCS intervention areas. The increase in utilization for PNC services is greater than that for home delivery by trained attendant. This may be because women who had labour during the night might find it convenient to deliver the child with attendance by the TBAs residing in the neighbourhood. But irrespective of who conducted the delivery, the mother would receive a PNC from the SK as soon as she is informed by the family members.

Very few studies have assessed the impact of community level interventions on equity and utilization of maternal health care. In this study the use of DiD and PSM provides a robust estimate of the impact of the intervention. However, one of the major limitations in using the DiD is that, it assumes the trends are parallel before and after the start of the intervention. It also assumes that differences between groups are attributed to the intervention rather than the impact of unmeasured factors. We were restricted to select only two comparison districts for the three intervention districts, instead of one for each. This was due to logistic and resource constraints. It needs to be mentioned here that if the interventional was designed as a cluster randomized trial, the impact of the intervention could be examined better.

## Conclusion

Addressing inequity in the utilization of maternal health services is essential to expedite the progress towards the MDG 5 target. It is important for those countries which are aiming to achieve the MDG 5 that they should focus on interventions that are effective and benefit the poor. In this study, use of maternal health care services was higher and was pro-poor in the intervention areas compared to the national average (22). Proper implementation of the programme and scaling it up in a larger number of districts would require rigorous monitoring of the intensive intervention activities. These would enable the achievement of the desired level of impact on utilization and equity. To sustain the equity in maternal health care utilization, the IMNCS programme needs to continue providing free home based services.

## Competing interests

The authors declare that they have no competing interests.

## Authors’ contributions

ZQ conceived the study and developed its design. ZQ and MNU prepared the initial draft of the manuscript, and revised it as per anonymous reviewers’ comments. ZQ and MNU conducted the analyses. TQ contributed to the review of literature. MC and HEN contributed to the initial draft of the manuscript. TE advised on analyses and design. All authors have read and approved the final manuscript.

## Supplementary Material

Additional file 1: Table S1 Activities of BRAC CHW of IMNCS Programme. Click here for file
