# Supplementary material for: “Can community level interventions have an impact on equity and utilization of maternal health care” – Evidence from rural Bangladesh
Source: Int J Equity Health. 2013 Apr 2;12:22. doi: 10.1186/1475-9276-12-22 (PMC3620556; doi:10.1186/1475-9276-12-22)
Supplement: Additional file 1: Table S1 — Activities of BRAC CHW of IMNCS Programme. [file 1475-9276-12-22-S1.docx]

Annex Table 1: Activities of BRAC CHW of IMNCS Programme.

| Each SS is responsible for 150 households and visits them working five days a week, covering 7 to 8 households every day. SS’s major activities include:  - counsels women on family planning, sell medicines and contraceptives (pills and condoms), and refer them to secondary care in case of contraceptive related complications,   - identify and visit pregnant mothers to advise them and their family members on ANC and other care needed during pregnancy and birth and informs about danger signs , delivery complications - motivate them for having at least one ANC from medically trained providers (MTP) . - accompany pregnant mothers for immunization against TT, - assist NHW during delivery, and give misopostral tablets (400 mg) to mothers as prophylactic treatment for post partum haemorrhage (PPH) - accompany mothers to the facility in case care is need for delivery complications - visit mothers and neonates until 42 days after delivery and counsels mothers about postpartum contraception. |
| --- |
| Each NHW is responsible for 300 households. Her major activities include :   - visiting the mother during last trimester at certain intervals and provides knowledge about delivery complications and birth preparedness plan - provide normal home delivery care to - regularly visit mothers until 42 days after delivery. |
| Each SK usually supervises 10 SS and 5 NHW and also visits 18-20 households per day. SK’s major activities include:   - provides monthly ANC to the mothers identified by the SS or by herself during the household visits and helps mothers to maintain necessary information in health card. - motivate the mothers for having at least one ANC from medically trained providers MTP). |
